# Supplementary material for: Broad variation in response of individual introns to splicing inhibitors in a humanized yeast strain
Source: bioRxiv. 2023 Nov 13:2023.10.05.560965. Originally published 2023 Oct 5. Preprint. [Version 2] doi: 10.1101/2023.10.05.560965 (PMC10592967; doi:10.1101/2023.10.05.560965)
Supplement: Supplement 1 [file media-1.zip › Hunter_et_al_Supplemental_Materials/Hunter_etal_FigS3G.pdf]

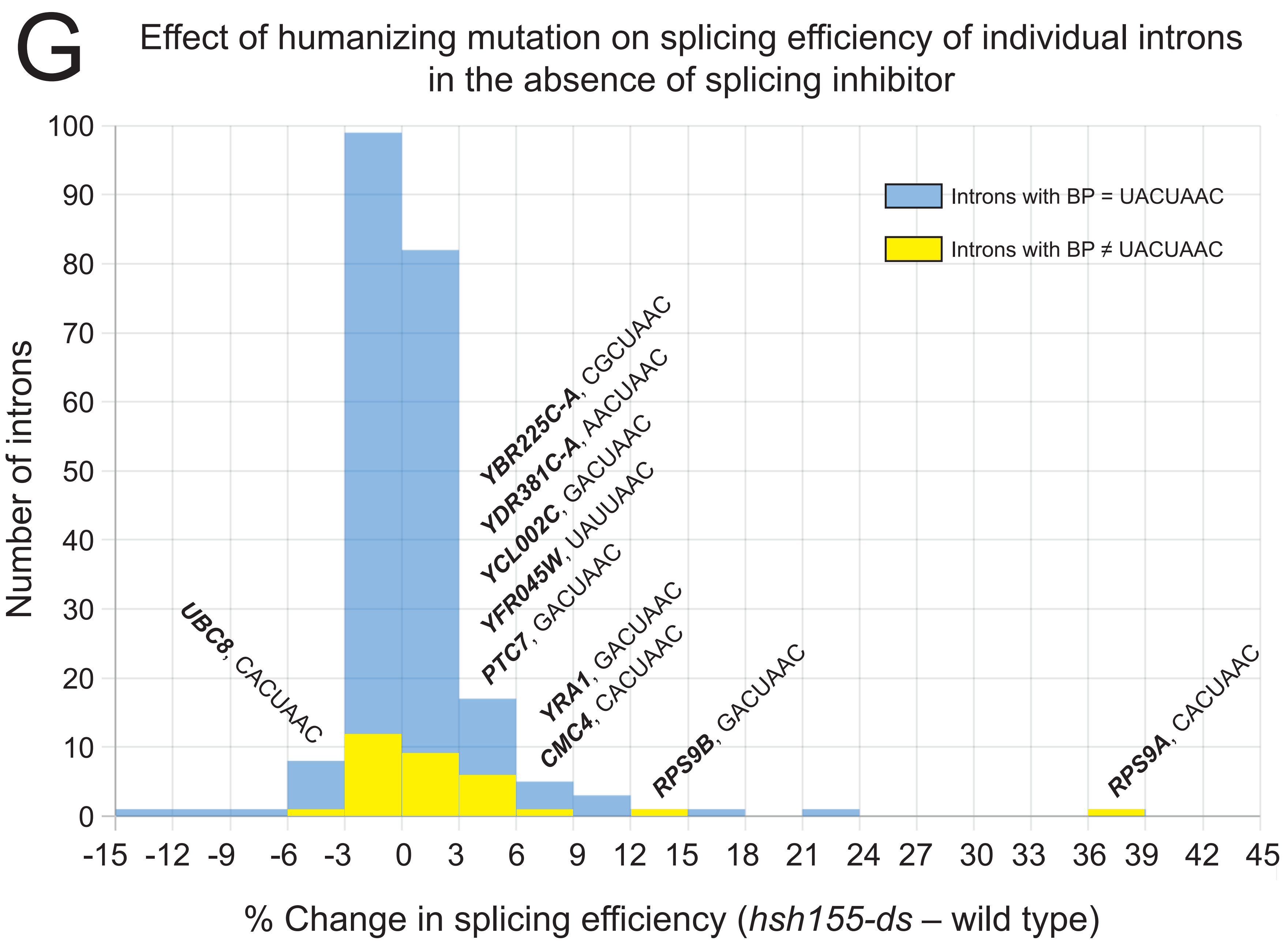

Hunter et al (Ares) Fig S4G
