# Supplementary figures and images for: Broad variation in response of individual introns to splicing inhibitors in a humanized yeast strain
Source: bioRxiv. 2023 Nov 13:2023.10.05.560965. Originally published 2023 Oct 5. Preprint. [Version 2] doi: 10.1101/2023.10.05.560965 (PMC10592967; doi:10.1101/2023.10.05.560965)

A

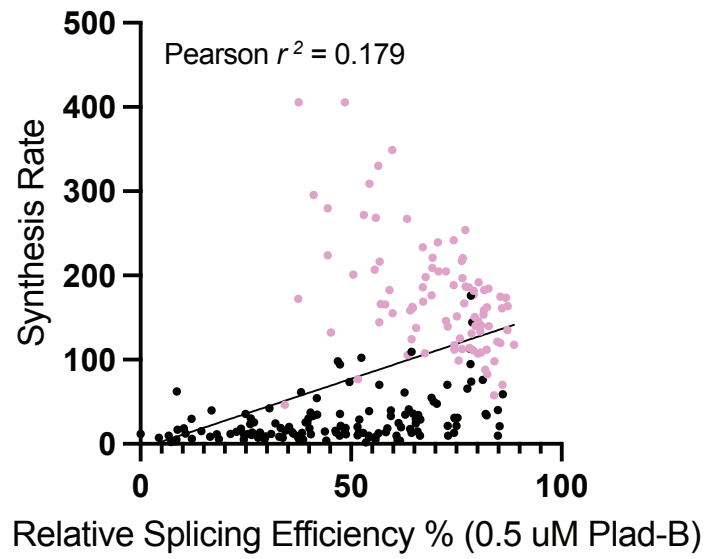

B

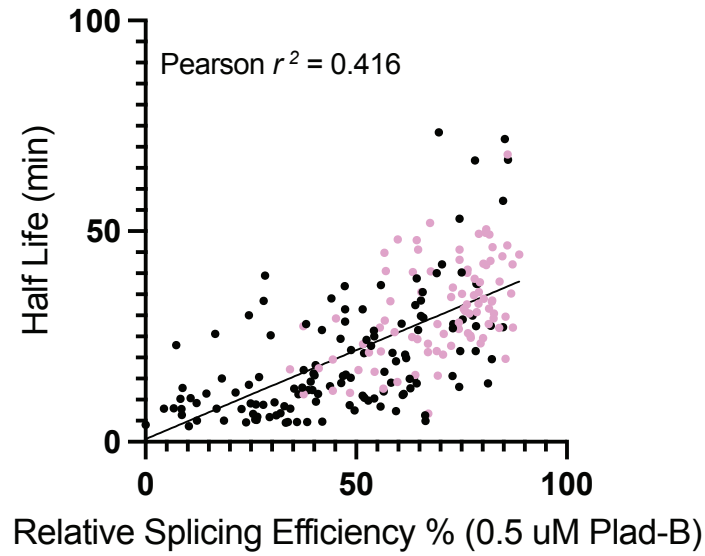

C

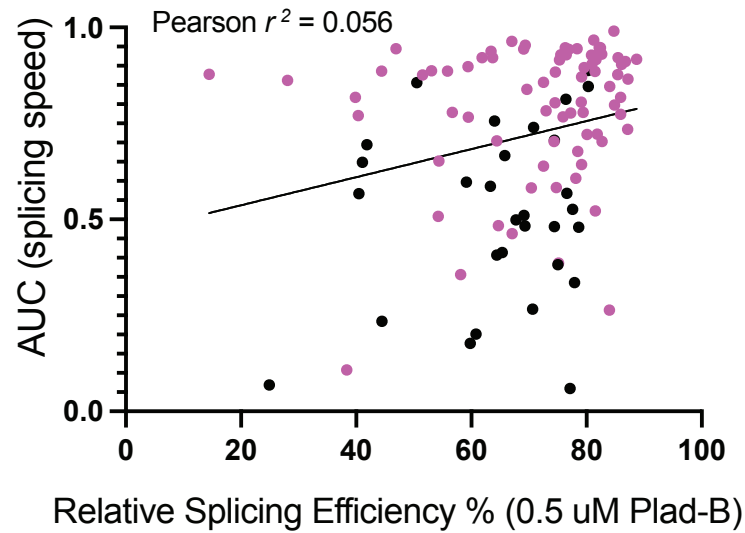

Supplement: Supplement 1 [file media-1.zip › Hunter_et_al_Supplemental_Materials/Hunter_etal_FigS4.pdf]

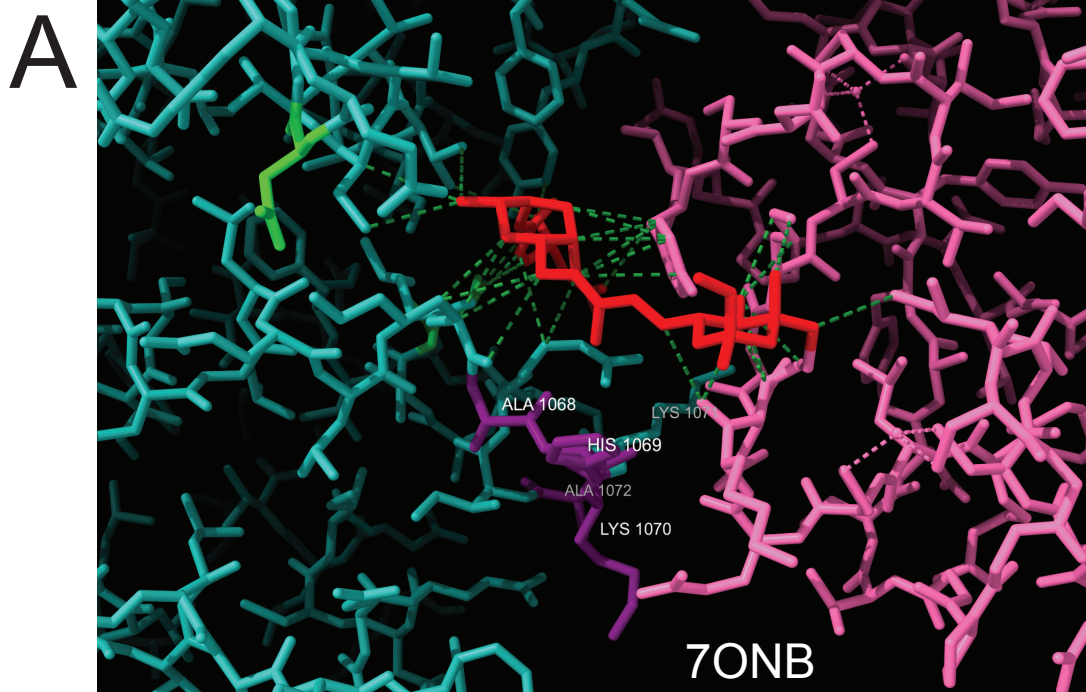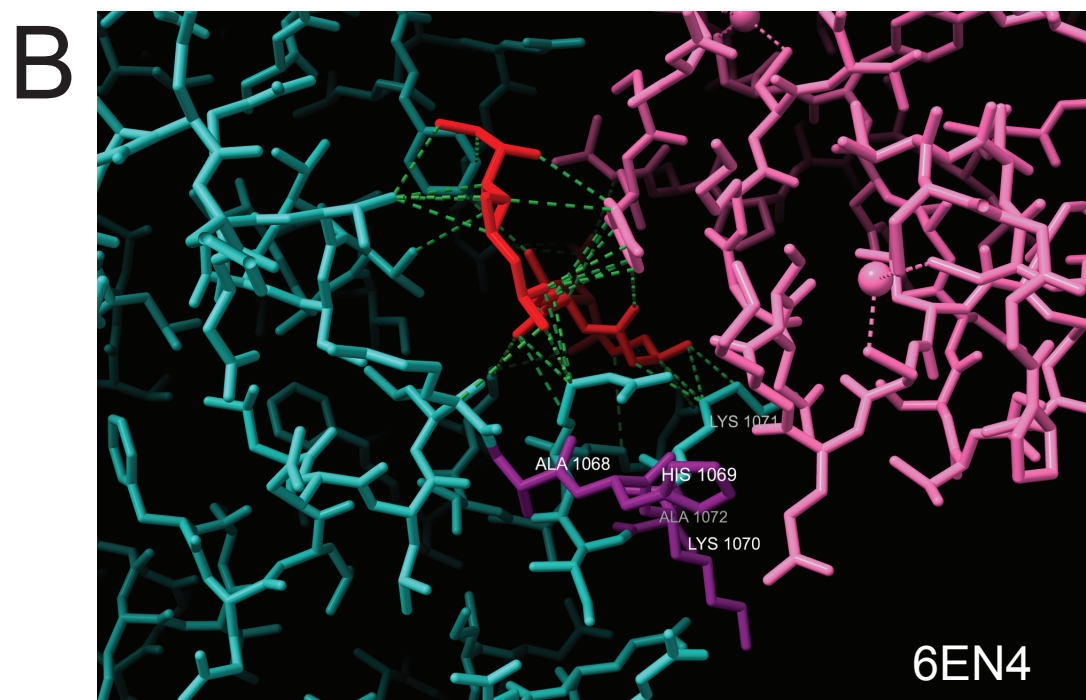

**C**

|       |      |       |      |
|-------|------|-------|------|
| human | 1068 | AHKKA | 1072 |
| yeast | 737  | STNKE | 741  |

Supplement: Supplement 1 [file media-1.zip › Hunter_et_al_Supplemental_Materials/Hunter_etal_FigS1.pdf]

**A** *hsh155-ds* + 0.5  $\mu$ M Plad-B

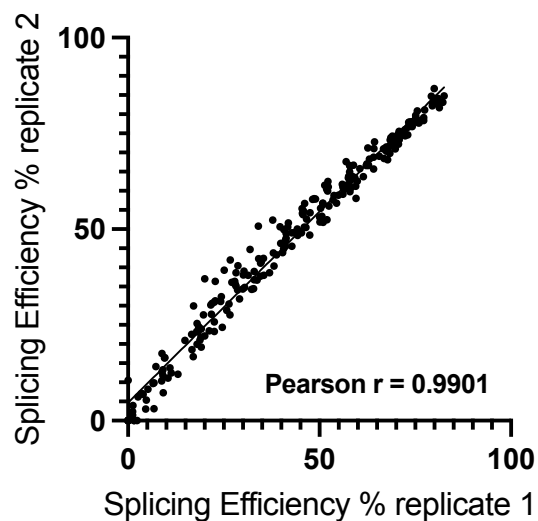

**B** *hsh155-ds* + 5  $\mu$ M Plad-B

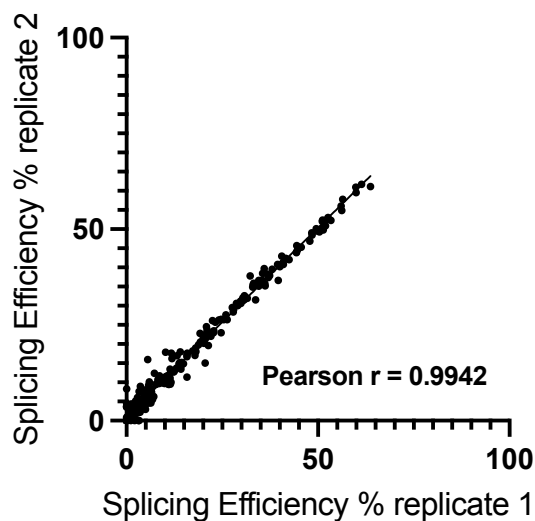

**C** *hsh155-ds* + 5  $\mu$ M Thail-A

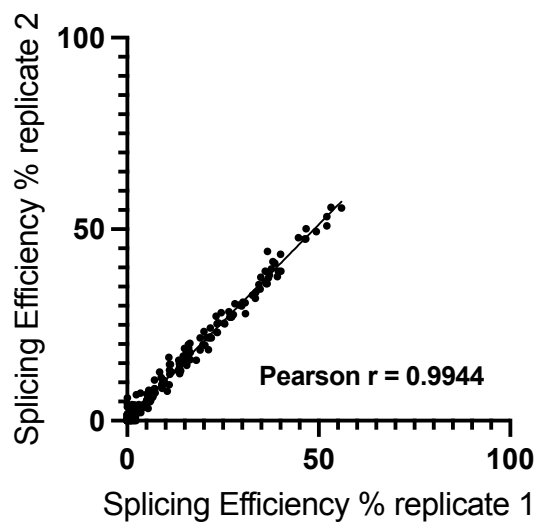

**D** *hsh155-ds* + DMSO

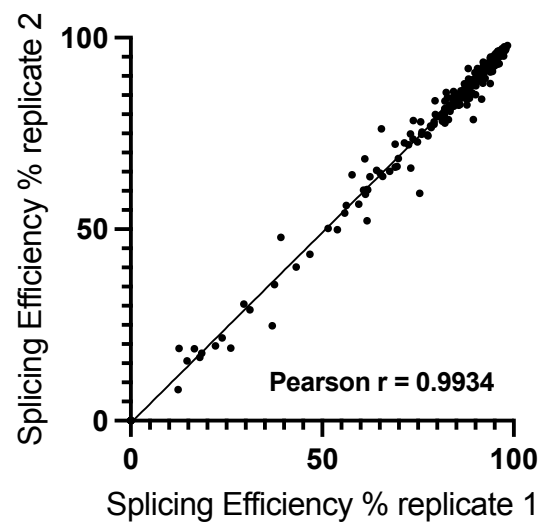

**E** WT HSH155 + 5  $\mu$ M Plad-B

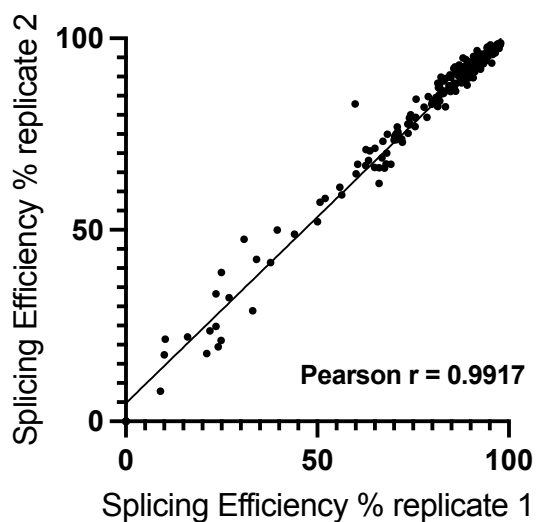

**F** WT HSH155 + DMSO

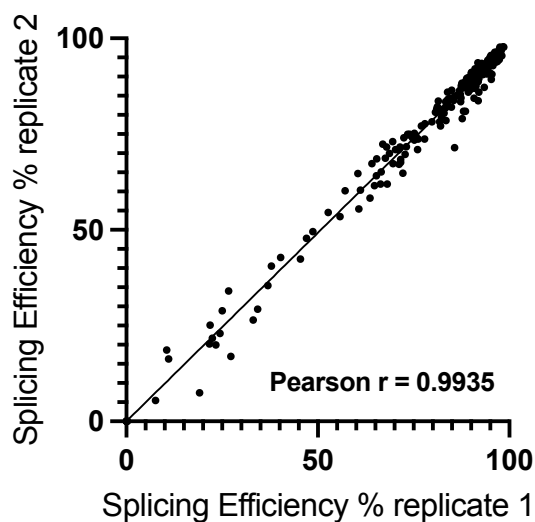

Supplement: Supplement 1 [file media-1.zip › Hunter_et_al_Supplemental_Materials/Hunter_etal_FigS3A-F.pdf]

# A

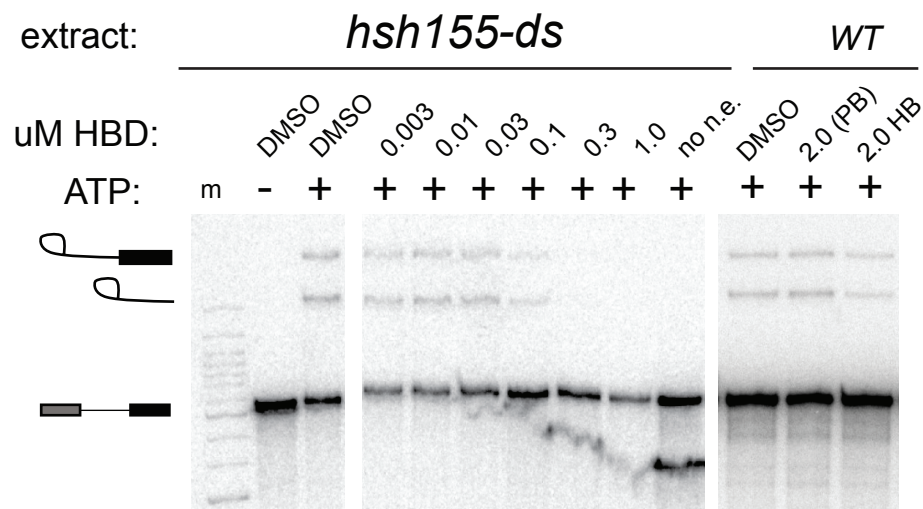

# B

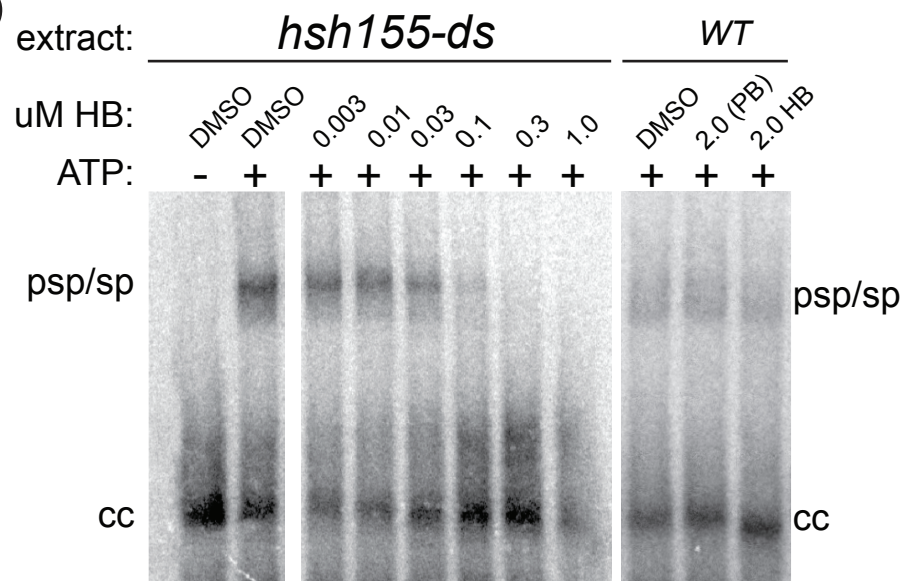

# C

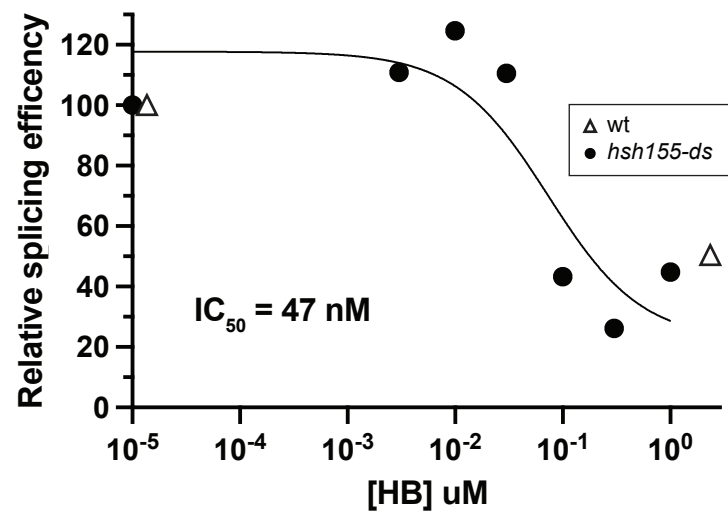

# D

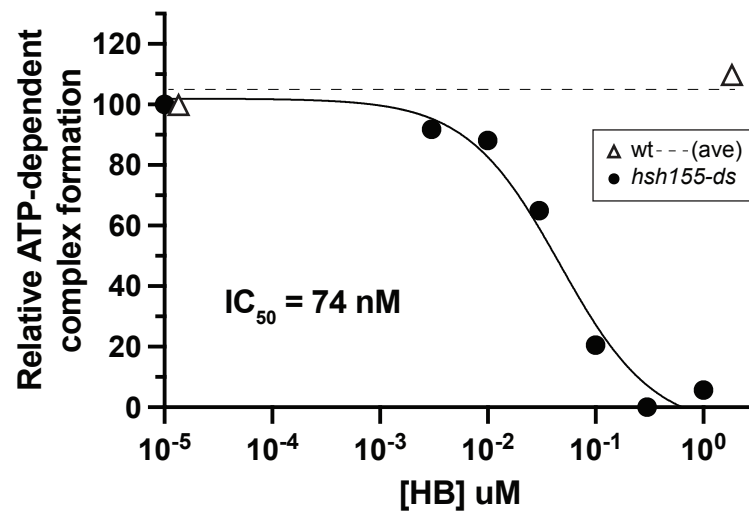

Supplement: Supplement 1 [file media-1.zip › Hunter_et_al_Supplemental_Materials/Hunter_etal_FigS2A-D.pdf]
